# Barriers in the Uptake and Delivery of Preconception Care: Exploring the Views of Care Providers

**DOI:** 10.1007/s10995-016-2089-7

**Published:** 2016-07-16

**Authors:** Hafez Ismaili M’hamdi, Sabine F. van Voorst, Wim Pinxten, Medard T. Hilhorst, Eric A. P. Steegers

**Affiliations:** 1 Department of Medical Ethics and Philosophy of Medicine, Erasmus University Medical Center Rotterdam, P.O. Box 2040, 3000 CA Rotterdam, The Netherlands; 2 Division of Obstetrics and Prenatal Medicine, Department of Obstetrics and Gynaecology, Erasmus University Medical Center Rotterdam, P.O. Box 2040, 3000 CA Rotterdam, The Netherlands; 3 Faculty of Life Sciences and Medicine, Hasselt University, 3500 Hasselt, Belgium

**Keywords:** Preconception care, Barriers, Uptake, Delivery, Preconception caregiver, Qualitative study

## Abstract

*Objectives* To examine health care professionals’ views of their role and responsibilities in providing preconception care and identify barriers that affect the delivery and uptake of preconception care. *Methods* Twenty health care professionals who provide preconception care on a regular basis were interviewed using semi-structured interviews. *Results* We interviewed twelve community midwives, three General Practitioners, three obstetricians, one cardiologist specialized in congenital heart diseases and one gastroenterologist.We identified four barriers affecting the uptake and delivery of preconception care (PCC): (1) lack of a comprehensive preconception care program; (2) limited awareness of most future parents about the benefits of preconception care, hesitance of GP’s about the necessity and effectiveness of PCC; (3) poor coordination and organization of preconception care; (4) conflicting views of health care professionals on pregnancy, reproductive autonomy of patients and professional responsibility. *Conclusion* We have identified four barriers in the uptake and delivery of preconception care. Our findings support the timely implementation of a comprehensive program of PCC (already advocated by the Health Council of the Netherlands) and increasing awareness and knowledge of PCC from care providers and future parents. We emphasize the need for further research on how organizational barriers lead to suboptimal PCC and how interdisciplinary collaboration and referral can lead to optimally tailored intervention approaches.

**Electronic supplementary material:**

The online version of this article (doi:10.1007/s10995-016-2089-7) contains supplementary material, which is available to authorized users.

## Significance

*What is already known about this subject?* Despite persistent adverse pregnancy outcomes and even though the benefits of preconception care have been established, the uptake and delivery of preconception care remain low. Health care professionals play an important role in the uptake and delivery of preconception care.

*What this study adds?* This study identifies barriers perceived by health care professionals. These barriers need to be addressed to improve the uptake and delivery of preconception care.

## Introduction

An increasing amount of research links fetal development with perinatal morbidity and mortality as well as the development of chronic diseases in later life (Gluckman et al. 2008; Jaddoe et al. 2014). Many risk factors for perinatal mortality and morbidity and associated diseases in adulthood are already present during the periconception period—the period before and shortly after conception—(Steegers-Theunissen et al. 2013). Targeting the periconceptional period opens opportunities to prevent later risks. Preconception care (PCC)—care and advice given before pregnancy—offers such an opportunity as it is offered before risk factors can exert negative effects on the developing fetus. A substantial body of evidence supports the benefits of PCC interventions on pregnancy outcomes (Jack et al. 2008; Shannon et al. 2013a; Temel et al. 2014; van der Zee et al. 2011) and influenced national and international recommendations and guidelines for the uptake and delivery of PCC (de Jong-Potjer 2011; Freda et al. 2006; Johnson et al. 2006) Most recommendations endorse the use of a standardized risk assessment which includes both medical and non-medical risks. (Temel et al. 2015; Williams et al. 2006).

Despite persistent adverse pregnancy outcomes and although the benefits of PCC have been established, the delivery and uptake of PCC remain low. In 2007, in response to the relatively high perinatal mortality and morbidity rates in the Netherlands, the Dutch Health Council published an advisory report entitled ‘Preconception care: a good beginning.’ The report emphasizes the importance of introducing a PCC program that is initiated and coordinated by the government. (Health Council of the Netherlands 2007) Guidelines for general practitioners and midwives (de Jong-Potjer 2011) as well as risk assessment instruments have been developed (Landkroon et al. 2010), and the Dutch government recognized the importance of introducing PCC as a standard component of perinatal care (Vos et al. 2016). Despite these recommendations, no comprehensive PCC program has been introduced and only few healthcare professionals are currently delivering PCC (van Voorst et al. 2016).

Healthcare professionals who deliver PPC (e.g., community midwives, general practitioners (GPs), obstetricians and other medical specialists) have the potential to significantly influence the uptake of PCC (de Weerd et al. 2002; Shannon et al. 2013b). But even though primary care setting, hospital setting, community outreach programs and youth health centers all offer opportunities to address and offer PCC (Tuomainen et al. 2013), healthcare professionals do not systematically discuss the availability and benefits of PCC in such settings (Mazza et al. 2013a, b; van Voorst et al. 2016).

The views held by those who provide PCC in different clinical settings influence the way in which they engage in PCC activities, discuss PCC with, and deliver PCC to future parents. A better understanding of the views of PCC providers regarding their role and responsibility towards PCC may help explain why the uptake is low.

The aim of this study is thus to explore the views, identify the barriers and provide recommendations to optimize the uptake and delivery of PCC.

## Methods

We conducted a qualitative interview with healthcare professionals who provide PCC in the Netherlands. As PCC is implemented on a small scale and there is no overview of where it is delivered, a convenience sample was selected for this interview study. The sample consisted of GPs, midwives, and specialists who deliver PCC on a regular basis (for our purposes defined as having delivered PCC at least 5 times in the previous year). The selected midwives delivered PCC on a weekly basis in midwifery practices. All selected GPs offered PCC in an opportunistic way.

We included specialists, who deliver specialist PCC, in order to compare whether their views differ from those of GPs and midwives who deliver regular PCC.

The familiarity with their patients offers them opportunities to discuss PCC at strategic moments, such as the removal of an IUD. All selected GPs offered PCC in opportunistic way.

Both GPs and midwives were selected from the list of participants of the ‘Healthy Pregnancy 4 All’ study (van Voorst et al. 2015); a study that evaluates the effectiveness of a preconception care program in urban and rural multi-ethnic communities from 14 municipalities in the Netherlands. In the ‘Healthy Pregnancy 4 All’ study, midwives and GPs were recruited to deliver PCC to requesting patients, thereby automatically making them eligible for our study by fulfilling the inclusion criterion of having delivered PCC at least 5 times within the previous year.

Specialists affiliated to the same university hospital as the authoring team and known to deliver hospital based PCC were invited to participate. Included specialists comprised of gynecologists, gastrointestinal specialist and cardiologists, all working at the Erasmus Medical Center Rotterdam, which delivers care to a multi-ethnic urban population. As university hospital employees, these specialists are involved in more complex PCC cases, sometimes after referral from GPs, midwives, or other specialists.

Semi structured interviews were performed using a questionnaire. In developing the questionnaire we carefully attended to the form and content of the questions. The form of the questionnaire was based on the Theoretical Domains Framework developed by Michie et al. (2005). This framework has been developed to enhance understanding of behavior change processes amongst health care professionals, which is an important determinant for the success of the clinical implementation of evidence-based practice such as PCC in the healthcare domain. It consists of a list of consensus-based theoretical domains (e.g., caregivers’ knowledge, skills, motivation and goals), which are essential for achieving a successful evidence-based implementation. Structuring our questions according to these domains enabled us to systematically identify the limiting factors for the delivery and uptake of PCC. That is, this framework offered the opportunity to link PCC barriers perceived by participants to a specific domain known to affect the uptake and delivery of healthcare. For each domain, sample questions were provided to evaluate implementation (see Table 1).

**Table 1:** Form: based on the theoretical domain framework developed by Michie et al

Domain	Questions
Knowledge	Are you familiar with the Dutch preconception guidelines? What do you think about the current organization of PCC? Is it feasible for you to perform your task as a preconception caregiver? How effective do you think PCC is? Do you think the goals of PCC are attainable?
Skills	How and with what aim do you ask the future parents about their medical and obstetric history? Other domains as well [Informative, directive (paternalistic), deliberative, shared decision making (Emanuel and Emanuel 1992)] What problems have you encountered when asking about the medical and obstetric history and how did you try to solve them? Can you give an example of such a problem? (And how were you able to solve the problem)
Social/professional role and identity	Do you encounter situations in which you think pregnancy should be postponed or discouraged because of the social economic conditions? Can this lead to a tension between your personal convictions and professional responsibility? (es.g. Personally I would advice against it however as a professional I feel obliged to advise and counsel)
Beliefs about capabilities	What problems have you encountered when delivering PCC in general? What problems have you encountered when asking about the medical and obstetric history and how did you try to solve them? How do you deal with the fact that working conditions can be hard to change, even if it is better for the health of the future parents and child?
Beliefs about consequences	Are you optimistic about the likelihood of tobacco, alcohol and drugs cessation? Do you think the current organization of PCC is adequate to help you solve the problems you encounter? How does the fact that these conditions (working conditions/social economic position) are hard to modify influence your delivery of PCC?
Motivation and goals	How valuable is PCC? Do you subscribe the goals of PCC and do they motivate you to do your job as a preconception caregiver? Does the social economic situation alter your motivation or goals when delivering PCC?
Memory, attention and decision processes	How much preparation do you need to deliver a preconception consultation and is it in balance with the perceived reward? (Reward can be a good consultation, health benefits for the future parents or monetary reward)
Environmental context and resources	What do you think about the current organization of PCC? Is it adequate for you to perform your task as a preconception caregiver? (Is there sufficient time and are there sufficient resources to perform your tasks as a preconception caregiver?)
Social influences	Do you feel sufficiently recognized valued in your work as a preconception caregiver by your patients and your peers?
Emotion regulation	Do you encounter situations in which you think pregnancy should be postponed or discouraged because of the medical or obstetric history? Can this lead to a tension between your personal convictions and professional responsibility? (E.g. Personally I would advice against it however as a professional I feel obliged to advise and counsel)
Behavioral regulation	What do you think about the current organization of PCC? Is it adequate for you to perform your task as a preconception caregiver
Nature of the behavior	Do you encounter any problems and what would help to overcome these problems?

The content of the questions was based on the Dutch guideline for GPs. (de Jong-Potjer 2011). This is a broad guideline for general comprehensive PCC that describes several risk domains that should be covered during preconception consultations for couples from the general public. This guideline includes the assessment of medical and obstetrical history, genetic risks, life style risks (including tobacco, alcohol and drug use, and risk exposure at work), genetic disorders, and socioeconomic factors (see Online Resource 1). We incorporated all these risk domains in our questionnaire.

To ensure consistency, only one interviewer (HI) conducted the interviews. Interviews had a duration of approximately 45 min. The semi structured interview format ensured that the preselected items were discussed but allowed to deviate from the interview format to explore new themes that were considered to be relevant by participants. The interviews were audiotaped and transcribed ad verbatim. All participants’ details were removed and the transcripts were de-identified to protect confidentiality.

Three authors (HI, WP, and MH) read the transcriptions independently from each other and subsequently discussed content to identify and compare the key barriers to PCC. The participants’ responses were classified using a deductive thematic method of analysis, in which the framework provided domains to organize the barriers mentioned by participants. Microsoft Excel software was used to organize these barriers.

## Results

Twelve community midwives, three general practitioners, three obstetricians, one cardiologist specialized in congenital heart diseases and one gastroenterologist were interviewed. The community midwives and GPs interviewed deliver general preconception consultation services, which cover the risk domains mentioned in the Dutch guideline for PCC. All the interviewed midwives and GPs indicated that they use ‘Zwangerwijzer’, a validated PCC questionnaire. The online version of Zwangerwijzer allows to generate an overview of the respondents risk profile. The interviewed midwives and GPs use this risk profile to deliver PCC as effectively and efficiently as possible. Only the GPs offered PCC opportunistically (i.e. when women request removal of an intrauterine device). Midwives indicated that opportunistic offers of PCC do not suit the midwifery because parents-to-be rarely visit a midwifery before conception. The interviewed specialists’ consultations typically aim to address complex medical issues that expose the patient and her future child to substantial health risks. All interviewed participants were aware of the Dutch guideline of PCC and shared the view that the delivery of PCC is of upmost importance when preparing for pregnancy. They also shared the view that despite this importance, the uptake of PCC remains disappointingly low.

The participant’s answers in combination with the domains from Michie’s framework provided the identification of four barriers that affect the uptake and delivery of preconception care: (1) lack of a comprehensive PCC program; (2) limited awareness of most future parents about the benefits of preconception care, hesitance of GP’s about the necessity and effectiveness of PCC; (3) poor organization and coordination of PCC; and (4) health care professionals’ conflicting views on pregnancy, reproductive autonomy of patients and professional responsibility.

## Lack of a Comprehensive PCC Program

The lack of a centrally coordinated and comprehensive offer of PCC (that is the lack of a PCC program in which the content of PCC is standardized) was raised as an important cause of the unfamiliarity with, and low knowledge of PCC amongst future parents. This unfamiliarity was thought to be the main reason for the low uptake of PCC. In addition, the low uptake of PCC also makes it difficult for healthcare professionals to develop a routine and build experience in the delivery of PCC.

(Knowledge, belief about capabilities) “Due to the low uptake, the frequency with which we do preconception consultations is low. Therefore we lack the opportunity to develop experience and routine in delivering PCC.” (Midwife)

All participants expressed the concern that future parents who would benefit the most from PCC are the ones who are the hardest to reach. Participants specifically identified future parents with low socioeconomic status, people living in poverty or deprived neighborhoods and non-western immigrants as hard to reach groups.

(Beliefs about capabilities) “PCC is simply unknown to a lot of people, especially to those who would benefit the most…I think that the people who would benefit the most are those who smoke, drink and are obese and live in deprived neighborhoods.” (Obstetrician)

(Beliefs about capabilities) “Especially people with a low SES perceive that they should only start seeking care once they are pregnant. The fact that they can optimize their health before pregnancy is unknown to them.”(Obstetrician)

Delivery of PCC is perceived to be time consuming because it is a new form of care and because of the substantial amount of risk factors that should be addressed during a consultation. Interviewed GPs and medical specialists indicated that they have insufficient time to deliver PCC. This lack of time was partly due to the fact that consultations are time consuming and partly because of competing preventive care which also needs to be delivered. Participants also indicated that future parents were not always willing to invest the required time and effort to adequately prepare for pregnancy.

(Environmental context and resources) “I often have to use all the time available to address the patient’s medical questions, so the time to ask about the desire to have children or to discuss PCC is lacking… Because of time and resource constraints, PCC has to compete with other preventive care. That may also be a barrier.”(GP)

(Beliefs about capabilities, beliefs about consequences) “I would like to see my patients invest more time in following my advice. It takes time to follow the advice I give them, like changing their medication or visiting another medical specialist for a check up. When I ask them to come see me again in three months they sometimes are reluctant to do so because they want to get pregnant as quickly as possible.” (Obstetrician)

Midwives perceived the current lack of a fee (no financial compensation) in combination with the labor-intensiveness as a barrier to deliver PCC.

(Environmental context and resources, motivation and goals) “The preconception consultation is very time consuming and we do not get paid for it.” (Midwife)

## Care Providers’ and Future Parents’ Lack of Knowledge of Preconception Care

Participants indicated that the future parents’ as well as healthcare professionals’ perceptions about PCC are important determinants for the uptake and delivery of PCC. The lack of familiarity with and knowledge of PCC of future parents and caregivers were perceived as barriers. GP’s in particular were somewhat hesitant to deliver PCC because, according to them, it is a time consuming form of care that still has to prove to be effective.

(Knowledge) “My patients’ knowledge about their health and about pregnancy is generally limited. They do not experience the need for PCC. This is a barrier for them to seek PCC.” (GP)

(Knowledge)“There is still a lot of uncertainty surrounding PCC. I am in favor of preventive healthcare interventions however I don’t know how evidence based some PCC interventions are…. excluding folic acid supplementation, tobacco and alcohol cessation and a good diet” (GP)

(Knowledge, Social/professional role and identity, memory attention and decision processes) “PCC is a relatively new form of care and, I think, not well known to many caregivers. And this unfamiliarity of caregivers with PCC is reflected in the amount of future parents seeking PCC.” (Midwife)

## Poor Organization and Coordination of Preconception Care

The proper delivery of PCC can be challenging because perinatal risk factors are multifactorial. Risk assessment and the subsequent timely referral to the appropriate caregiver are paramount. GPs and specialists indicated that in general, the healthcare professionals’ ability to timely identify all the different healthcare needs of future parents needs improvement. Women who have a substantial risk to experience complications during pregnancy, are too often not referred to the appropriate specialist. The inability of non-specialists to identify patients who need tailored PCC was perceived as a barrier. In addition, the poor or even lack of communication between the different healthcare disciplines that offer PCC was also identified as a cause for insufficient referral of patients to the appropriate caregiver and perceived as a barrier.

(Social influences, beliefs about capabilities)“It is really important that patients are referred in time to the right caregivers which unfortunately doesn’t always happen… the communication between the different disciplines of PCC seems to be fragmented which makes the provided care suboptimal and less efficient.”(GP)

(Social influences, beliefs about capabilities)“In this hospital we have cardiologists who are specialized in managing congenital heart defects in young people, also during pregnancy. This includes delivering tailor-made PCC. A general cardiologist has less experience and expertise to provide this specific care. Although we encourage the referral of these patients to a hospital that can provide the required care, this unfortunately doesn’t happen enough.”(Cardiologist specialized in congenital heart diseases)

(Social influences, beliefs about capabilities) “Midwives, GP’s and obstetricians have insufficient expertise about inflammatory bowel disease to provide adequate care for patients who have a desire to become pregnant. However, these patients who should be seen by me or one of my colleagues are too often not referred to us.” (Gastroenterologist)

## Ethical Barriers

The client’s or patient’s medical history or non-medical risks can lead to situations where healthcare professionals would advice to postpone pregnancy or even advise against it. However, healthcare professionals also want to respect the clients’ and patients’ right to autonomously choose when to become pregnant. An ethical dilemma can arise when a patient persists in her wish to conceive against the advice of the healthcare professional and in spite of medical grounds to postpone or stall pregnancy. The tension between personal beliefs about pregnancy and the well being of the future child on the one hand and the professional responsibility to provide the best care possible for patients while respecting the reproductive autonomy of the future parents on the other hand, was perceived as a barrier. However, all participants stated that they would, under no circumstance, forfeit their professional responsibility to provide care for their patients once they are pregnant.

(Social/professional role and identity, emotion regulation, motivation and goals) “A barrier is that sometimes you personally think that, considering the patient’s medical history, it might be better for her not to get pregnant. However as a caregiver my task is to advise and guide her regardless of my personal view.”(GP)

(Social/professional role and identity, emotion regulation, motivation and goals)“Sometimes you see cases where for example the patient lives in squalid conditions, has financial debts or is bedridden. These are difficult situations. I would ask my patient how she would take care of her child once it is born. The hope is that through discussion you can give an honest view of how difficult it would be for her to raise a child in her situation and perhaps convince her to postpone or give up her desire to have a child. However, if she decides to become pregnant I will advise and guide her as good as possible.”(Obstetrician)

## Discussion

The results of our study suggest that there are four barriers to the uptake and delivery of PCC. 1) Due to a lack of a comprehensive PCC program, the future parents’ and caregivers’ limited familiarity with and knowledge of PCC is perpetuated. This barrier is particularly worrisome because the groups who would benefit the most from PCC such as future parents with a lower SES and non-western future parents, are the ones who are the hardest to reach with PCC. 2) Most future parents are unaware of the benefits of PCC. GP’s are hesitant about the necessity and effectiveness of PCC. 3) Perinatal risk factors are multifaceted. It is important that future parents receive care from the proper caregiver. GPs and medical specialists expressed the concern that too often patients who need specialized care are not referred or are referred too late to them. 4) There are situation where women trying to conceive are well advised to postpone pregnancy, but may choose to become pregnant regardless. Even when participants thought that choosing to become pregnant for a patient was the wrong choice, all participants clearly expressed that they would favor their professional responsibility and the patients’ reproductive autonomy over their own personal views.

This study shows that there is an unfamiliarity with and lack of knowledge about PCC. The participants of this study indicate that both the unfamiliarity and lack of knowledge towards PCC are reasons why the uptake towards such care remains low. The low uptake due to lack of knowledge about PCC was also observed by Hosli et al. (2008) and van der Zee et al. (2013). The GPs indicate that time and resource constraints as well as competing preventive care were barriers to deliver PCC. This was also observed by Mazza and colleagues (Mazza et al. 2013a, b). Our study draws attention to the barriers that result from the lack of a comprehensive PCC program. This barrier was anticipated by the Dutch Health Council that advised to set up a governmentally initiated and coordinated program of PCC, sustaining that this approach will reach the greatest number of future parents and create the most favorable conditions for monitoring the effectiveness, efficiency and social consequences of PCC (Health Council of the Netherlands 2007). Unfortunately the advice to set up a PCC program has not yet lead to the implementation of a comprehensive and coordinated PCC program in the Netherlands.

Participants, especially the GPs and specialists, pointed out that even though timely referral of patients with complex medical and obstetric history to adequate caregivers is paramount, such patients are too often not referred or are referred too late.

We do stress the need for further studies that look into the ways in which these organizational barriers lead to suboptimal PCC delivery and into how interdisciplinary collaboration can result in optimally tailored PCC. However, because the inadequate referral of patients is an urgent matter we recommend the implementation of a PCC program as was suggested by the Dutch Health Council. We also support the inclusion of PCC in the academic curriculum of future healthcare professionals. We suggest hat the implementation of a PCC program and the inclusion of PCC in the curriculum of future caregivers will increase overall knowledge about, and awareness of, PCC in general, and will promote adequate referral of future parents with a complex medical history. Education about PCC should include evidence-based findings of research on PCC. This is of particular importance because, as our study shows, GP’s remain hesitant about the effectiveness and efficiency of PCC. This hesitation is a barrier for the (opportunistic) offering of PCC in healthcare settings.

Furthermore, efforts to train and educate caregivers should not end at graduation, especially in the case of PCC. The participating midwives pointed out that the low uptake of PCC reduces opportunities to gain the necessary experience in delivering PCC. This barrier was also identified by Heyes et al. (2004). In their study, they describe that barriers to provide PCC include a lack of contact with women planning to conceive. In addition, van Heesch et al. (2006) also reported that few midwives had received any training on PCC after qualifying in their discipline. They show that midwives seem willing to play an active role in the provision of preconception care in the future, but that there is a great need for continued training with practicing healthcare providers.

In some cases patients with complex medical conditions or with difficult financial and social problems do wish to become pregnant, even against the caregiver advices to postpone pregnancy. Caregivers can personally feel that these patients are making an incorrect decision when they insist on pregnancy. However, our results do not indicate that the caregivers’ personal considerations lead to a suboptimal uptake or delivery of PCC. Nevertheless, we recommend that the curriculum of PCC caregivers should include ethical education and guidance so that in practice caregivers will be more competent in dealing with these dilemmas.

### Strengths

Incorporating risk domains mentioned in the Dutch guideline preconception care and composing the questionnaire for this study according to the framework Michie and colleagues ensured quality and relevance of the questionnaire. Furthermore, given the fact that the participants in our study were all experienced in the delivery of PCC according to the Dutch guideline, they were ideally positioned to report on barriers on the uptake and delivery of PCC. Finally, the variety of disciplines in which the participants included in our study practiced allowed to identify barriers experienced in PCC as a whole. Ultimately, in accordance to the views of participants, PCC requires a multidisciplinary approach. This requires knowledge about barriers perceived by the whole ambit of healthcare professionals who deliver PCC.

### Limitations

The small number of participants, which is common in qualitative studies, limits the generalizability of our findings. However, interviews were conducted until saturation of responses was achieved. We do recommend the confirmation of our results by other studies.

## Conclusion

Our study has identified four barriers for the optimal uptake and delivery of preconception care. Given the explorative nature of our study, we recommend that further research is done to gain a better understanding of these barriers and to determine which barriers should be prioritized for intervention. In addition, we highlight the need for further research into ways in which organizational barriers lead to suboptimal PCC delivery and how interdisciplinary collaboration can result in optimally tailored intervention approaches.

However, the recommendation for further research should not hinder the introduction and integration of PCC as a government coordinated program since the benefits of PCC interventions such as folic acid supplementation, alcohol and tobacco cessation and the promotion of a healthy diet have provided sufficient evidence to be made a priority in healthcare.

## Electronic supplementary material

Below is the link to the electronic supplementary material.

Supplementary material 1 (DOCX 82 kb)
