# Supplementary material for: Barriers in the Uptake and Delivery of Preconception Care: Exploring the Views of Care Providers
Source: Matern Child Health J. 2016 Jul 16;21(1):21–8. doi: 10.1007/s10995-016-2089-7 (PMC5226984; doi:10.1007/s10995-016-2089-7)
Supplement: Supplementary file 1 — Supplementary material 1 (DOCX 82 kb) [file 10995_2016_2089_MOESM1_ESM.docx]

| **Online Resource 1** | |  |
| --- | --- | --- |
| *Content: based on the Dutch guideline of general practitioners on preconception care* | |  |
| **Domain** | **Questions** | |
| Preconception care in general | - What do you think about the current organization of PCC? Is it adequate for you to perform your task as a preconception caregiver? Do you encounter any problems and what would help to overcome these problems? - How valuable is PCC? Do you subscribe the goals of PCC and do they motivate you to do your job as a preconception caregiver? - How effective do you think PCC is? Do you think the goals of PCC are attainable? - Do you feel sufficiently recognized and valued in your work as a preconception caregiver both by your patients and your peers? | |
| Medical and obstetric history | - In what way and with what aim do you ask the future parents about their medical and obstetric history? (Informative, directive (paternalistic), deliberative, shared decision making^23^) - What problems have you encountered when asking about the medical and obstetric history and how did you try to solve them? - Can you give an example of such a problem? (And how were you able to solve the problem) - Do you encounter situations in which you think pregnancy should be postponed or discouraged because of the medical or obstetric history? Can this lead to a tension between your personal convictions and professional responsibility? (E.g. Personally I would advice against it however as a professional I feel obliged to advise and counsel) - Do you think the current organization of PCC is adequate to help you solve these problems? What kind of adjustments to PCC would ameliorate your capability to deal with these problems? | |
| Genetic disorders | - How and with what aim do you ask the future parents about genetic disorders? - Does the difficulty of the subject matter change your role as a caregiver? (E.g. from informative to directive?) | |
| Exposures at work | - How and with what aim do you ask the future parents about their working conditions? - How do you deal with the fact that working conditions can be hard to change, even if it is better for the health of the future parents and child? | |
| Socioeconomic factors | - How and with what aim do you ask the future parents about their social economic positions? - How does the fact that these conditions are hard to modify influence your delivery of PCC? - Do you encounter situations in which you think pregnancy should be postponed or discouraged because of the social economic conditions? Can this lead to a tension between your personal convictions and professional responsibility? (E.g. Personally I would advice against it however as a professional I feel obliged to advise and counsel) - Does the social economic situation alter your motivation or goals when delivering PCC? | |
| Tobacco, alcohol and drugs | - How and with what aim do you ask about tobacco, alcohol and drugs use? - Are you optimistic about the likelihood of tobacco, alcohol and drugs cessation? | |
